# Maternal loss-of-function of Nlrp2 results in failure of epigenetic reprogramming in mouse oocytes

**DOI:** 10.21203/rs.3.rs-4457414/v1

**Published:** 2024-06-04

**Authors:** Zahra Anvar, Michael D. Jochum, Imen Chakchouk, Momal Sharif, Hannah Demond, Alvin K. To, Daniel C. Kraushaar, Ying-Wooi Wan, Simon Andrews, Gavin Kelsey, Ignatia B. Veyver

**Affiliations:** Baylor College of Medicine; Baylor College of Medicine; Baylor College of Medicine; Baylor College of Medicine; Babraham Institute; Baylor College of Medicine; Baylor College of Medicine; Baylor College of Medicine; Babraham Institute; Babraham Institute; Baylor College of Medicine

**Keywords:** Subcortical maternal complex, Oocyte, Imprinted genes, DNA methylation

## Abstract

**Background:**

NLRP2 belongs to the subcortical maternal complex (SCMC) of mammalian oocytes and preimplantation embryos. This multiprotein complex, encoded by maternal-effect genes, plays a pivotal role in the zygote-to-embryo transition, early embryogenesis, and epigenetic (re)programming. The maternal inactivation of genes encoding SCMC proteins has been linked to infertility and subfertility in mice and humans. However, the underlying molecular mechanisms for the diverse functions of the SCMC, particularly how this cytoplasmic structure influences DNA methylation, which is a nuclear process, are not fully understood.

**Results:**

We undertook joint transcriptome and DNA methylome profiling of pre-ovulatory germinal-vesicle oocytes from *Nlrp2*-null, heterozygous (Het), and wild-type (WT) female mice. We identified numerous differentially expressed genes (DEGs) in Het and *Nlrp2*-null when compared to WT oocytes. The genes for several crucial factors involved in oocyte transcriptome modulation and epigenetic reprogramming, such as DNMT1, UHRF1, KDM1B and ZFP57 were overexpressed in Het and *Nlrp2*-null oocytes. Absence or reduction of *Nlrp2*, did not alter the distinctive global DNA methylation landscape of oocytes, including the bimodal pattern of the oocyte methylome. Additionally, although the methylation profile of germline differentially methylated regions (gDMRs) of imprinted genes was preserved in oocytes of Het and *Nlrp2*-null mice, we found altered methylation in oocytes of both genotypes at a small percentage of the oocyte-characteristic hyper- and hypomethylated domains. Through a tiling approach, we identified specific DNA methylation differences between the genotypes, with approximately 1.3% of examined tiles exhibiting differential methylation in Het and *Nlrp2*-null compared to WT oocytes.

**Conclusions:**

Surprisingly, considering the well-known correlation between transcription and DNA methylation in developing oocytes, we observed no correlation between gene expression differences and gene-body DNA methylation differences in *Nlrp2*-null versus WT oocytes or Het versus WT oocytes. We therefore conclude that post-transcriptional changes in the stability of transcripts rather than altered transcription is primarily responsible for transcriptome differences in *Nlrp2*-null and Het oocytes.

## Background

The processes of transcription, translation, mRNA and protein stability and degradation, and epigenetic reprogramming are highly regulated during oogenesis and early embryo development. In mice, previously quiescent oocytes in primordial follicles resume transcription in the early post-natal period, and transcription continues during oocyte growth until it is globally repressed in fully grown germinal vesicle (GV) stage oocytes [1], but oocytes retain cytoplasmic stores of transcripts and proteins encoded by maternal effect genes (MEGs) that are essential for further oocyte maturation, progression and completion of meiosis, fertilization, and the earliest stages of embryo development. The newly fertilized embryo is also initially transcriptionally quiescent and depends entirely on the actions of these stored maternal transcripts and proteins. The length of the embryo’s transcriptional quiescence correlates with the oocyte’s size and the amount of cytoplasm it contains, which varies between species [2, 3]. During the maternal-to-zygotic transition (MZT), the stored maternal mRNAs are degraded, and zygotic genome activation (ZGA) takes place, marking the timepoint when the embryo begins to transcribe its own biparentally inherited genome [4, 5].

In mice, transcription drives de novo DNA methylation [6, 7] and directly affects DNA methylation [8]. Approximately 10 days after birth, DNA methylation establishment is initiated by the DNMT3A/L complex in mouse growing oocytes and is completed by the GV stage, at around day 21 when oocytes have about 40% global DNA methylation [7, 9, 10]. The global DNA methylation landscape of oocytes is unique and differs profoundly from that of somatic cells. In oocytes, which are non-replicative cells, DNA methylation is primarily targeted to actively transcribed units, resulting in a bimodal methylome, consisting of broad hypomethylated domains (HypoDs) and hypermethylated domains (HyperDs). The latter are mostly associated with gene bodies and encompass intragenic CpG islands (CGI) [11, 12]. Other genomic features that acquire methylation in oocytes are maternal germline differentially methylated regions (gDMRs) of imprinted genes [13]. The mono-allelic methylation of these gDMRs is necessary for their parent-of-origin monoallelic expression after fertilization and throughout the lifespan [14]. Genetic or epigenetic disturbances in the expression of imprinted genes cause imprinting disorders [15]. Therefore, the necessity for epigenetic marking of gDMRs is an important reason why oocyte DNA methylation is essential for successful development [16].

In mammals, a subset of MEGs code for proteins of the subcortical maternal complex (SCMC), a multiprotein complex in the cytoplasm of oocytes and outer cells of preimplantation embryos. This complex plays a vital role in various essential cellular processes during the MZT [17–19]. In mice, NLRP5, KHDC3, OOEP, TLE6, NLRP2 and PADI6 are key members that stabilize the complex [20–22]. Of those, NLRP5, OOEP, TLE6 and PADI6 are crucial for development beyond the two-cell stage because their absence from oocytes and embryos before MZT leads to female sterility [22–25], whereas the absence of other SCMC proteins, like NLRP2 and KHDC3, causes milder and variable effects, including delayed preimplantation development and decreased fecundity [21, 26]. Other proteins associated with the complex have since been found, and other components are probably still to be discovered, consistent with the SCMC’s high molecular weight (between 669 and 2000 kDa) [27–32]. A human SCMC has also been identified [33] and maternal loss of function of some of the genes that encode SCMC proteins result in embryonic lethality, biparental complete hydatidiform mole (BiCHM), and recurrent pregnancy loss [34–38].

NLRP2 is a member of the SCMC, the maternal loss of which has been associated with a wide array of reproductive and offspring phenotypes in both humans and mice. *Nlrp2* knockdown in mouse GVs causes embryonic arrest between the two-cell and eight-cell stages [29]. We previously showed that female mice lacking *Nlrp2* are subfertile and have adverse reproductive outcomes. Their offspring have variable phenotypes that can include early embryonic loss, growth restriction, delayed development, and congenital anomalies [21]. The embryonic phenotype was exacerbated during *in vitro* culture, where embryos from *Nlrp2*-null females failed to form blastocysts [39]. This underscores that the embryonic phenotype in mice is worse under *in vitro* conditions. We also showed in these mice that maternal loss of *Nlrp2* alters the transcript abundance of hundreds of genes in superovulated metaphase-II (MII) oocytes, potentially contributing to the abnormal reproductive phenotypes and reduced viability of embryos [40]. Although we found abnormal DNA methylation at a few imprinted loci in affected offspring of *Nlrp2*-null females [21], we did not yet examine if the transcriptome changes in MII oocytes were also associated with locus-specific or globally altered DNA methylation. It has also been shown that maternal loss-of-function variants of *NLRP2* in humans causes pregnancy loss and loss of methylation at maternally methylated imprinted gDMRs. This can manifest in a range of imprinting disorders, including multilocus imprinting disturbance (MLID), transient neonatal diabetes mellitus (TNDM), Beckwith-Wiedemann syndrome (BWS), Silver-Russell syndrome (SRS), as well as signs of growth restrictions and developmental delays in probands that were not consistent with any specific diagnosis [41, 42]. These combined mouse and human data indicate that NLRP2 plays a role in epigenetic regulation, supporting a potential broader role for the SCMC in this process. How loss of cytoplasmic NLRP2 and other SCMC proteins impacts epigenetic programming and reprogramming, particularly DNA methylation, remains poorly documented. Our previous work in human showed a genome-wide loss of DNA methylation in oocytes and pre-implantation embryo caused by maternal loss-of-function of *KHDC3L* [43]. Giaccari and colleagues recently found that maternal loss of *Padi6* in mice causes genomic hypermethylation, decreased transcript abundance of ZGA genes, and increased transcript abundance of maternal decay genes in 2-cell embryos, that were isolated from superovulated pregnant females. In addition, superovulated MII oocytes from *Padi6*-null females had significant transcriptome and subtle methylome changes. They also found disrupted intracellular localization of the maintenance DNA methyltransferase 1, DNMT1, and its co-factor UHRF1 in GV oocytes, zygotes and 2-cell embryos from superovulated *Padi6*-null females [44]. Taken together, these data indicate that studies on the mechanisms by which these maternal-effect mutations cause the adverse reproductive outcomes should be focused on oocytes of these mice.

In the present study we profiled the transcriptome and methylome of GV oocytes from *Nlrp2*-null, Het and WT females, at the developmental stage immediately after the completion of oocyte transcription and DNA methylation establishment. Analysis at the GV-oocyte stage also avoids the confounding effects due to superovulation. To achieve this, we used genome and transcriptome sequencing (G&T-seq), in which physical separation of RNA and DNA from single cells or small numbers of cells, allows paired analysis of DNA methylation profiles and transcriptomes from the same pools of cells.

## Results

### Absent or reduced Nlrp2 alters ovarian follicle maturation and the transcriptomes of GV oocytes.

**Figure 1:**
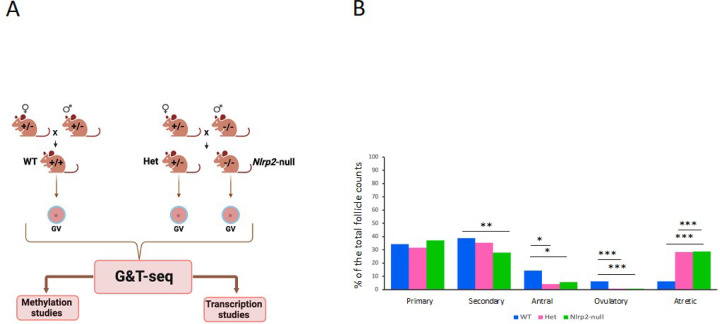
**a** Experimental Design. GV oocytes were isolated from WT, Het and *Nlrp2*-null females. Each library (biological replicate) was prepared from a pool of 60–80 GV oocytes isolated from both ovaries of one female. **b** Follicle distribution in ovaries from WT, Het and *Nlrp2*-null mice at P24. We counted five biological replicates of follicles in both ovaries per genotype. Follicle distributions are represented as percentages of total follicular counts (*** = *P*<0.001, ** = *P*<0.01, * = *P*<0.05)

We collected GV oocytes from 26–28 days-old WT, Het and *Nlrp2*-null female mice to study their DNA methylome and transcriptome. Oocytes were collected in pools of 60–80 oocytes per animal from both ovaries combined, with each pool from a single mouse serving as one biological replicate (Fig. 1a and Supplementary Fig. 1). To avoid potential reproductive effects of maternally inherited null alleles in Het females, we only included oocytes from Het females who carry a paternally inherited null allele. There was no difference in the number of retrieved oocytes between the three genotypes (One-Way ANOVA: *p* = 0.66) (Supplementary Table 1). However, we noted that isolated GVs from *Nlrp2*-null tended to degenerate faster compared to the other two genotypes, indicating that total absence of *Nlrp2* reduces oocyte viability. We also counted follicle distribution of both ovaries of five females each per genotype at postnatal day 24 (P24). Compared to WT, *Nlrp2*-null ovaries contained fewer secondary (*p* = 0.003), antral (*p* = 0.04) and ovulatory follicles (*p* <0.001), but more atretic follicles (*p* < 0.001), and Het ovaries contained fewer antral (*p* = 0.016), ovulatory (*p* < 0.001) but more atretic (*p* < 0.001) follicles (Fig. 1b) (Multiple comparisons with Bonferroni, Supplementary Table 2). This indicates that absence or reduction of NLRP2 impairs follicle maturation dynamics.

**Figure 2:**
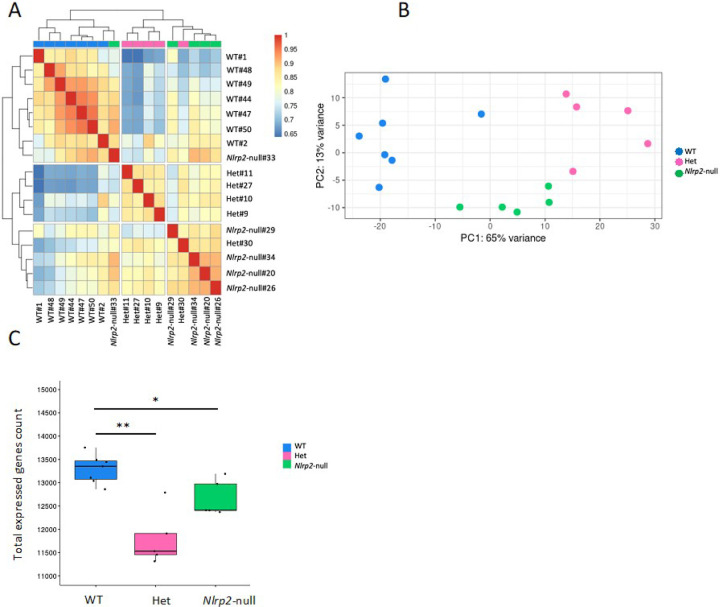
**a** Correlation matrix for WT, Het and *Nlrp2*-null GV oocyte RNA-seq libraries. This heatmap shows the pairwise Euclidean distances between RNA-seq libraries after applying a variance stabilizing transformation (VST) to the gene count data. Each cell corresponds to the calculated distance between two RNA-seq libraries, providing a visual representation of library similarity, where a value of 1 is an ideal correlation. **b** PCA Plot of Genotype Clustering. Dots in the plot represent an RNA-seq library colored by genotype (WT: blue, Het: pink, *Nlrp2*-null: green). The three genotypes exhibit clear separation along the principal components. X-axis: Principal Component (PC) 1 and Y-axis: PC2. **c** Box plot showing total number of expressed genes in oocytes from WT, Het and *Nlrp2*-null. The mean number of expressed genes for each library (represented by dots) and the median for each group are indicated (** = *P*<0.01; * = P<0.05).

We next profiled the transcriptome of seventeen GV oocyte pools, derived from seven WT, five Het and five *Nlrp2*-null females. Upon running the standard analysis pipelines, we observed some overexpressed unannotated genes that appeared as novel splices that do not occur at canonical splice junctions. These could result from trans-splicing between genes, the presence of variable amounts of unprocessed RNA, or DNA contamination in the cDNA libraries. To ensure a rigorous analysis, we excluded the approximately 4% of reads associated with these splices and retained only those aligning entirely across known splice junctions. The detailed metrics of the RNA sequencing (RNA-seq) experiments are in Supplementary Table 3. Each RNA-seq library had an average read count of 45,606,512 (range: 23,036,578–118,110,366). An euclidean distance-based correlation matrix of gene counts showed a correlation between samples (Fig. 2a) and principal component analysis indicated a clear separation of oocyte transcriptomes by genotype (Fig. 2b). After selecting only those genes that were covered by at least one read in at least one sample, we retained 15,622 genes, with an average of 12,671 per library. Of these, 9,906 were in common between all libraries. Of note, fewer expressed genes were detected in Het and *Nlrp2*-null oocytes compared to WT oocyte (Fig. 2c) (Supplementary Table 3) (One-Way ANOVA: *p* < 001, Wilcoxon test: *p* = 0.002 for Het versus WT and *p* = 0.03 for *Nlrp2*-null versus WT).

**Figure 3:**
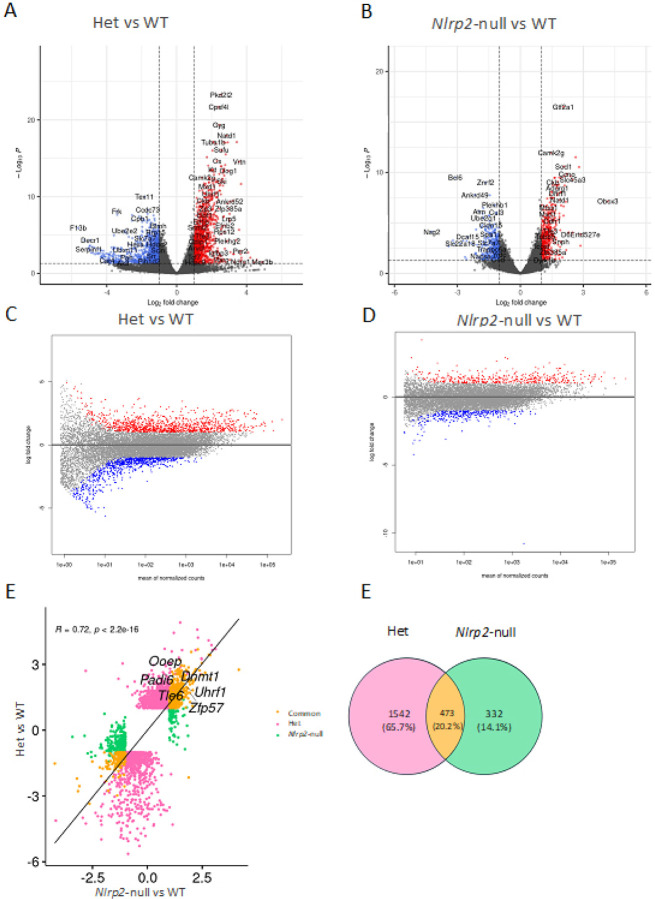
**a, b** Volcano plots of DEGs between Het and WT (**a**) or *Nlrp2*-null and WT (**b**) oocyte pools. Note: we removed *Nlrp2* from **b** for better visualization of the other DEGs but refer to supplementary Figures 2 for volcano plot that includes *Nlrp2*(**Red:** overexpressed genes; blue: underexpressed genes; gray: genes without significant expression difference; vertical dashed lines: Log2FC cutoff; horizontal dashed lines: significance level). **c** and **d,** MA plots of Log2 fold changes (y-axis) over mean expression levels (x-axis) in Het (**c**) and *Nlrp2*-null (**d**) versus WT oocyte pools (with same colors for DEGs). **e** Scatter plot displaying DEGs in Het compared to WT against DEGs in *Nlrp2*-null compared to WT oocyte pools (Orange: common DEGs between Het versus WT and *Nlrp2*-null versus WT comparisons; pink: DEGs from Het to WT comparison; green DEGs from *Nlrp2*-null to WT comparison). Note *Nlrp2* is removed from the scatter plot to have a better visualization of other DEGs. **f** Venn diagram of overlap between DEGs identified from Het versus WT and *Nlrp2*-null versus WT.

Differential gene expression was determined by DESeq2 analysis on the combined oocyte pools from each genotype followed by filtering for genes with |Log2FC| > 1 and adjusted *p* value < 0.05. It is important to recognize for this analysis that fully mature GV oocytes at the surrounded nucleolus (SN) stage, are transcriptionally quiescent cells that store previously transcribed RNAs. Therefore, the identification of differentially expressed genes (DEGs) is not solely indicative of differences in transcriptional activity between genotypes but could also reflect altered RNA abundance that arose from differences in post-transcriptional processes that influence RNA stability and degradation. Thus, for this analysis the term “overexpressed DEGs” refers to more abundant transcripts while “underexpressed DEGs” refers to less abundant transcripts. We found 2015 DEGs between Het and WT oocyte pools and 805 DEGs between *Nlrp2*-null and WT oocyte pools (Supplementary Tables 4 and 5, respectively). From the 2015 DEGs between Het and WT oocytes, 1113 were overexpressed and 902 were underexpressed in Het oocytes (Fig. 3a). From the 805 DEGs between *Nlrp2*-null and WT oocytes, 412 were overexpressed and 393 were underexpressed in *Nlrp2*-null oocytes (Fig. 3b and Supplementary Fig. 2). We then conducted Gene Ontology (GO) enrichment analysis to find the most enriched biological processes among the identified DEGs. Overexpressed DEGs in Het were enriched for transcription by RNA polymerase II (GO:0006366), translation (GO:0006412), DNA-templated transcription (GO:0006351), regulation of cellular biosynthetic process (GO:0031326) and nucleobase containing compound metabolic process (GO:0006139). Overexpressed DEGs in *Nlrp2*-null were enriched for regulation of gene expression (GO:0010468), prevention of polyspermy (GO:0060468), microtubule cytoskeleton organization (GO:0000226), in utero embryonic development (GO:0001701) and nervous system development (GO:0007399). Underexpressed DEGs in Het were enriched for organic acid metabolic process (GO:0006082), determination of left/right symmetry (GO:0007368), reciprocal meiotic recombination (GO:0007131), transmembrane transport (GO:0055085) and cilium movement (GO:0003341). The latter GO term is quite intriguing. The involvement of motile cilia in the detachment of cumulus cells from fertilized zygote has been demonstrated. Motile cilia, located on the apical surface of multi-ciliated cells in the epithelium of the oviduct, serve as the primary driver of oocyte and embryo towards the uterus for implantation. The absence of motile cilia results in female infertility [45]. Underexpressed DEGs in *Nlrp2*-null were enriched for aerobic respiration (GO:0009060), gamete generation (GO:0007276), mitochondrion organization (GO:0007005), RNA processing (GO:0006396) and apoptotic process (0006915). All these biological processes align with the recognized role of the SCMC in early development. (Supplementary Table 6 contains the output of the GO enrichment analysis for overexpressed and underexpressed DEGs in *Nlrp2*-null and in Het).

We next inspected MA plots which visualize DEGs between Het and WT oocytes (Fig. 3c) and between *Nlrp2*-null and WT oocytes (Fig. 3d) while also factoring in the average overall expression levels for each gene across both genotypes. We found that DEGs from both the Het to WT and the *Nlrp2*-null to WT comparisons are distributed across the entire range of expression levels (Fig. 3c and d). However, we noted that particularly in the Het to WT comparison, highly abundant genes are enriched for overexpressed DEGs and lowly abundant genes are enriched for underexpressed DEGs. This observation, coupled with the identification of lower overall expressed gene counts in oocytes of Het and *Nlrp2*-null mice, along with the impaired follicle maturation in those mice, implies that both Het and *Nlrp2*-null oocytes are distinctly different from WT oocytes. However, it is worth mentioning that fertilized Het oocytes give rise to viable healthy embryos, suggesting that the observed gene expression changes in Het oocytes do not impair their capacity for fertilization and early embryo development.

We next examined if overexpressed and underexpressed genes from the *Nlrp2*-null versus WT analysis change gene expression in the same direction as in the Het versus WT analyses (Fig. 3e, and supplementary Fig. 3). We found that the combined 2347 significant DEGs from the *Nlrp2*-null versus WT and Het versus WT analyses were generally expressed in the same direction in Het and *Nlrp2*-null oocytes (Fig. 3e and Supplementary Fig. 3), but that only a subset reached significance in one of these comparisons, including 1542 that were unique to Het versus WT and 332 that were unique to *Nlrp2*-null versus WT analyses. Notably, the expression of 473 DEGs was significantly altered across both comparisons (Fig. 3f), most of which (325) were overexpressed in both *Nlrp2*-null and Het oocytes compared to WT oocytes (Supplementary Table 7). Strikingly, the common overexpressed DEGs contain many genes that encode important epigenetic regulators, including ZFP57, DNMT1 and UHRF1, and genes that encode other proteins of the SCMC, including TLE6, OOEP and PADI6 (Fig. 3e). This highlights the involvement of NLRP2 in early development through these factors.

### Genome-wide oocyte-specific DNA methylation patterns are preserved in oocytes from Nlrp2-null, Het and WT mice.

**Figure 4:**
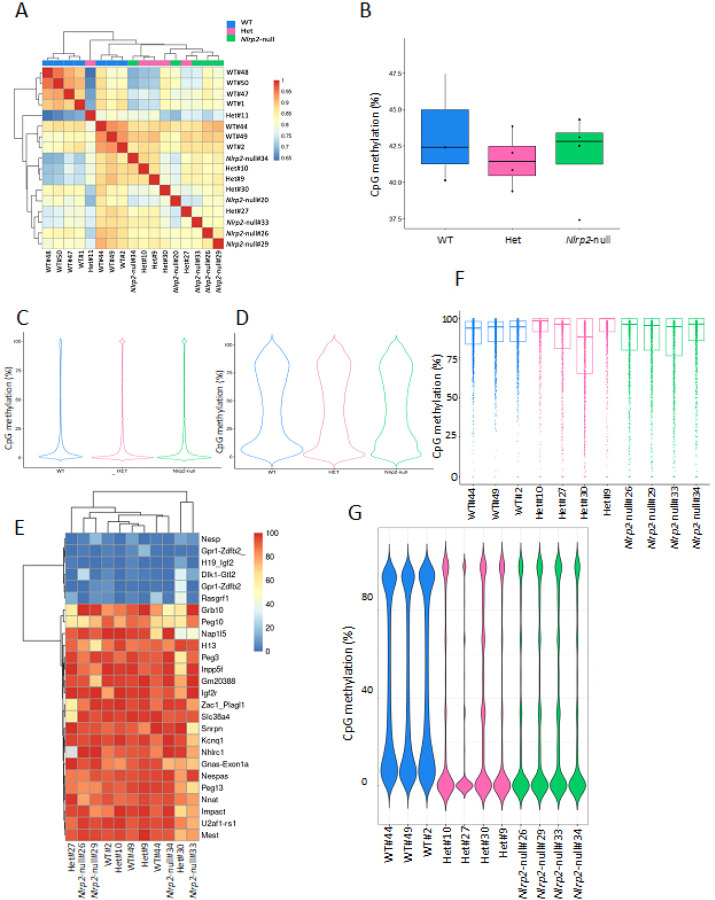
**a** Correlation matrix between PBAT libraries based on the methylation of CpG sites shows association between libraries at individual CpG sites. Distance metric is based on Manhattan distance and clustering method is Ward.D2. **b** Box plot of overall CpG methylation levels for each genotype. The mean CpG methylation levels for each oocyte pool (represented by dots) and the median for each group are indicated. **c, d** Violin plots of methylation levels of promoters (C) and gene bodies (**d**). Analysis was performed on 3 WT, 4 Het, and 4 *Nlrp2*-null oocyte pools. **e** Heatmap of methylation levels of maternal and paternal gDMRs in 3 WT, 4 Het and 4 *Nlrp2*-null oocyte pools (0–100 represents *%* methylation). The minimum coverage is 6 CpGs within the *Nhlrc1* maternal gDMR in the library *Nlrp2*-null#26. **f** Methylation levels of oocyte-specific CGIs in WT, Het and *Nlrp2*-null oocyte pools. Box plots show median (center line), upper and lower quartiles (box limits), 1.5X interquartile range (whsikers) and outliers (points). **g** Violin plot of genome-wide DNA methylation in 100-CpG tiles on selected pools of 3 WT, 4 Het, and 4 *Nlrp2*-null oocytes. These libraries exhibit the expected highly bimodal pattern, but with more variability in Het and *Nlrp2*-null oocyte pools.

Oocytes have unique DNA methylation features compared to somatic cells, an important one being that transcription drives the establishment of *de novo* DNA methylation in oocytes [10]. We therefore used the post-bisulfite adaptor tagging (PBAT) method with slight modifications [46, 47] to profile genome-wide DNA methylation on the same seven WT, five Het and five *Nlrp2*-null oocyte pools that were used for transcriptome profiling. After alignment and removal of duplicate sequence reads, we retained between 2,084,314 and 27,718,913 uniquely mapped reads per library for further analysis (Supplementary Table 8). We first observed that the CpG methylation profiles of all PBAT libraries are highly similar, with most of the pairwise correlations greater than 0.8. Moreover, the *Nlrp2*-null libraries and Het libraries cluster closer to each other than to WT libraries (Fig. 4a). Sequencing yielded 2,054,114 CpGs as the mean number of CpGs covered by at least 3 reads (3X coverage) in each PBAT library (Supplementary Table 8). However, to do a more rigorous analysis we wanted to exclude methylomes from oocyte pools with granulosa cell contamination and low read counts. As a proxy for granulosa cell contamination, we used methylation levels at X chromosome CpG islands, which is very low in oocytes, compared to granulosa cells [48], and only included methylomes from pools with an average X chromosome CpG island methylation level of < 13.5%. Additionally, we set up a cutoff for read counts (> 5,000,000 uniquely mapped reads). Applying these exclusion criteria resulted in a total of 11 oocyte methylomes for further analysis: three WT (WT#2, WT#44 and WT#49), four Het (Het#9, Het#10, Het#27 and Het#30) and four *Nlrp2*-null (*Nlrp2*-null#26, *Nlrp2*-null#29, *Nlrp2*-null*#33* and *Nlrp2*-null#34) (see also supplementary method and supplementary Figs. 4A- D).

The percentage of genome-wide CpG methylation (35.1–47.5%) and non CpG methylation (4.1–5.4% for CHG and 4–5.8% for CHH methylation) across all libraries were as expected for GV oocytes (Supplementary Table 8). The median CpG methylation was 42.3% in WT oocyte pools, 41.4% in Het oocyte pools, and 42.7% in *Nlrp2*-null oocyte pools, with no statistically significant differences between the genotypes (WT versus Het; T-test: *p* = 0.63, WT versus *Nlrp2*-null; T-test: *p* = 1) (Fig. 4b).

We also examined DNA methylation levels at all promoters and gene bodies. We found that the median methylation at promoters was significantly lower in Het (1.6%) and *Nlrp2*-null oocytes (2.1%) compared to WT oocytes (3.6%) (Tukey multiple comparisons, *p* < 0.001 for both) (Fig. 4c). Of the 14,089 promoters with desired 3X coverage, only 274 had different DNA methylation levels between genotypes (Supplementary Table 9). GO analysis of genes associated with these promoters showed that the most enriched biological processes were mRNA processing (GO: 0006397), blastocyst development (GO: 0001824) and blastocyst hatching (GO: 0001835).

When we quantified methylation levels over gene bodies, we saw no differences between *Nlrp2*-null (41.9%) and WT (41.8%) oocytes, but slightly higher methylation in Het oocytes (43.5%) compared to WT oocytes (Tukey test for multiple comparisons, *p* = 0.003) (Fig. 4d).

We next sought to comprehensively investigate the properties of various characteristic CpG methylation features in oocytes, including the methylation patterns and levels of gDMRs, CpG islands (CGIs), oocyte-specific CGIs, and the presence of bimodal DNA methylome, which can serve as indicators of fidelity of oocyte CpG methylation pattern.

We evaluated CpG methylation levels of gDMRs because of their importance in early development and genomic imprinting. We found that maternal gDMRs are highly methylated and paternal gDMRs are unmethylated in oocyte pools, with no difference between the three genotypes (Fig. 4e). Then, we investigated the methylation levels of CGIs in the oocyte pools. We had sufficient coverage to analyze DNA methylation of a subset of 11,720 CGIs of the total ~ 23,000 present in the mouse genome [14, 49]. Applying a threshold of q < 0.05 and methylation difference of 10%, we found that only six CGIs had differential methylation between WT and Het oocytes and only nine between WT and *Nlrp2*-null oocytes (Supplementary Table 10). We then assessed CpG methylation levels at the 2140 highly methylated oocyte-specific CGIs [14, 49] as an additional indicator of the expected methylation pattern of normal oocytes and found that all were highly methylated, irrespective of oocyte genotypes (Fig. 4f). Hence, even though Het and *Nlrp2*-null oocytes had more variable overall methylation patterns compared to WT oocytes, methylation at gDMRs and CGIs was not significantly impacted by reduced or lack of *Nlrp2*. This is not unexpected given the nature of these genomic elements, as any impact on their methylation is expected to cause a pronounced embryonic phenotype, which is inconsistent with outcomes in embryos conceived from *Nlrp2*-null females [21].

Next, to ascertain the bimodal DNA methylome of mouse oocytes, we employed an unbiased 100-CpG tiling approach, with each tile containing 100 consecutive CpGs. As shown in Fig. 4g, the analyzed 11 methylomes exhibit the expected bimodal DNA methylation pattern of oocytes, with the lower bump on the violin plot corresponding to regions with low methylation levels (perhaps HypoDs), and the upper bump corresponding to regions with high methylation levels (perhaps HyperDs). These results indicate similar global DNA methylation landscape in oocytes of the three genotypes.

within the *Nhlrc1* maternal gDMR in the library *Nlrp2*-null#26. **f** Methylation levels of oocyte-specific CGIs in WT, Het and *Nlrp2*-null oocyte pools. Box plots show median (center line), upper and lower quartiles (box limits), 1.5X interquartile range (whsikers) and outliers (points). **g** Violin plot of genome-wide DNA methylation in 100-CpG tiles on selected pools of 3 WT, 4 Het, and 4 *Nlrp2*-null oocytes. These libraries exhibit the expected highly bimodal pattern, but with more variability in Het and *Nlrp2*-null oocyte pools.

### Robust changes in DNA methylation at discrete loci in GV oocytes lacking Nlrp2.

**Figure 5:**
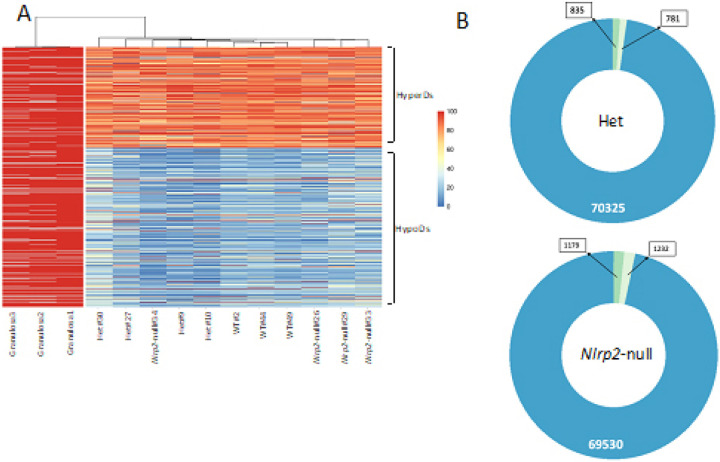
**a** Heatmap of distribution of HyperDs and HypoDs: HyperDs (upper cluster) and HypoDs (lower cluster) of selected oocyte pools (right 11 lanes) compared to granulosa cells (three left lanes) are shown (0–100 scale represents relative methylation levels). **b** Doughnut graphs of maintained and lost HyperDs and HypoDs in Het and *Nlrp2*-null oocyte pools. Each donut represents the 71,941 analyzed domains in this comparison. Colors and numbers indicate maintained domains (blue), lost HyperDs (dark green) and lost HypoDs (light green) in Het (top) and *Nlrp2*-null (bottom) oocytes.

The above quantitative analysis of 100-CpG tiles (Fig. 4g) indicates that the bimodal DNA methylation pattern representative of the presence of HyperDs/HypoDs is consistently observed in all 11 tested oocyte methylomes. However, this does not provide details on the number, distribution, and actual methylation levels of HyperDs/HypoDs. To address this, we examined the methylation levels of each individual HyperD or HypoD. We retained 71,941 HyperDs/HypoDs in our WT oocyte methylomes (out of originally reported 94,513 by Veselovska and colleagues [11]). The methylation levels of each of these domains were then measured (Fig. 5a). This analysis showed that all oocyte pools – irrespective of their genotypes – contain characteristic oocyte-specific HyperDs and HypoDs (Fig. 5a). To further confirm the specificity of this analysis, we also aligned methylation profiles from granulosa cells (which are not expected to be enriched for HyperDs and HypoDs) and confirmed the absence of HyperDs and HypoDs in those cells (left three lanes on Fig. 5a). (See also Supplementary method and supplementary Figs. 4A-4D). To further examine the distribution of HyperDs/HypoDs, assess whether specific location of HyperDs/HypoDs are maintained or lost, and if new ones emerged in Het and *Nlrp2*-null oocytes we set up a cutoff for HyperDs as average methylation ≥75% and for HypoDs as average methylation of ≤25%. We found that of the known HyperDs and HypoDs, 835 HyperDs and 781 HypoDs did not reach these thresholds in Het oocytes (Fig. 5b top) and that 1172 HyperDs and 1239 HypoDs did not reach these thresholds in *Nlrp2*-null oocytes (Fig. 5b bottom), which we interpreted those as lost HyperDs and HypoDs. This indicates that even though only a small fraction of HyperDs and HypoDs are lost from Het and *Nlrp2*-null oocytes, the overall structure and distribution of HyperDs and HypoDs, a signature feature of oocytes, is sensitive to maternal loss and haploinsufficiency of NLRP2.

**Figure 6:**
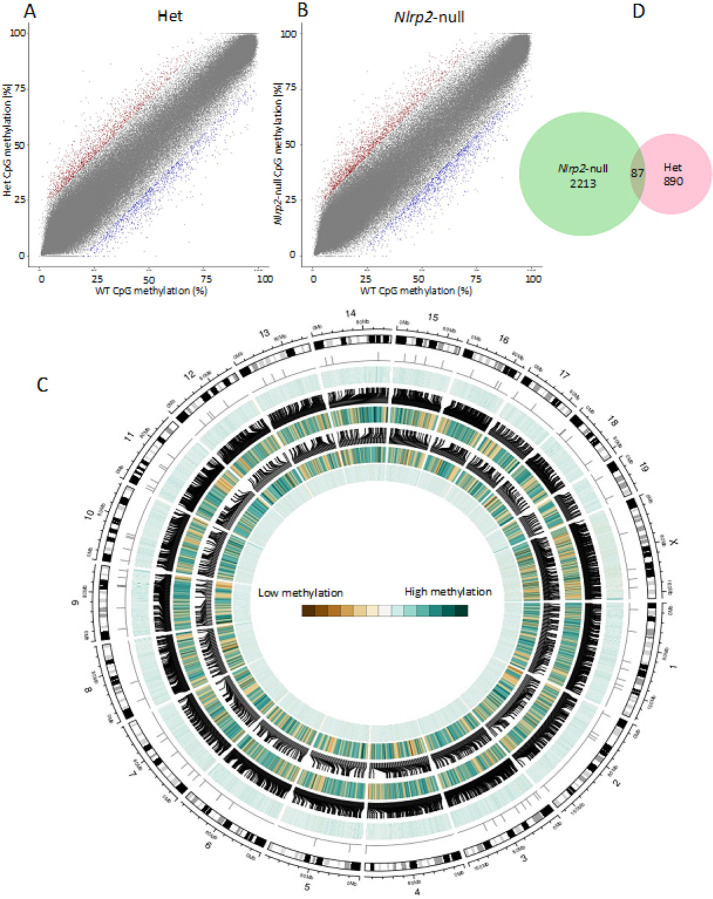
Scatterplots of average methylation values of 10 Kb tiles: (**a**) Comparison between Het (n = 4) and WT oocyte pools (n = 3), and (**b**) *Nlrp2*-null (n = 4) and WT oocyte pools (n= 3). Each dot is one tile. Red is hypermethylated in Het or *Nlrp2*-null and blue is hypomethylated in Het or *Nlrp2*-null compared to WT oocytes. **c** Circos graph comparing genome-wide methylation levels in *Nlrp2*-null versus WT and Het versus WT oocytes by chromosome. From outer to inner circle: Chromosomes number and size, G-banding pattern, 87 common DMRs, all informative tiles in *Nlrp2*-null versus WT in light blue, a brush indicating occupancy of DMRs out of all informative tiles in black, the 2300 DMRs identified in *Nlrp2*-null versus WT in brown to green, a brush indicating the occupancy of DMRs out of all informative tiles in black, the 977 DMRs identified in Het versus WT in brown to green, all informative tiles in Het versus WT in light blue (Brown to green represents low to high methylation differences). **d** Venn diagram of the number of unique and common DMRs between *Nlrp2*-null versus WT (green) and Het versus WT (pink) comparison.

We next sought to identify any generally differentially methylated regions (DMR) between Het and WT oocytes and between *Nlrp2*-null and WT oocytes. For this analysis we employed the adjacent 10-kb tiling approach, which yielded 252,972 informative tiles with 3X coverage in all 11 samples. A methylation difference of a tile was considered statistically significant by a chi square test corrected for multiple comparisons using the SLIM method (*q* < 0.01) and when the difference between two genotypes was ≥ 20%. Applying these criteria, we identified 378 hypomethylated and 599 hypermethylated DMRs in Het compared to WT oocyte pools (Fig. 6a) (Supplementary Table 11) and 907 hypomethylated and 1393 hypermethylated DMRs in *Nlrp2*-null oocytes compared to WT oocytes (Fig. 6b and Fig. 6c) (Supplementary Table 12). These DMRs were evenly distributed across all chromosomes (Fig. 6c), but we noted that *Nlrp2*-null and Het DMRs were more abundant in intergenic regions than in promoters, exons, or introns (one-way ANOVA, *p* = 0.01) (Supplementary Fig. 5). There were 87 DMRs in common between Het versus WT and *Nlrp2*-null versus WT comparisons, which were equally distributed across chromosomes (Fig. 6c and Fig. 6d). Although a gene maybe much larger than a DMR, which spans a 10 kb tile, we have identified interesting genes or clusters associated with these common DMRs. The first is the maternally imprinted *Gnas* gene, located in a locus with a highly complex imprinted expression pattern. It has been shown that the methylation status of the GNAS cluster can be used as a marker for oocyte quality [50] (Supplementary Fig. 7A). The second gene is *Jarid2*, encoding a cofactor of polycomb complex PRC2, which methylates histone mark H3K27 and is essential for embryonic stem cell differentiation and preimplantation development [51] (Supplementary Fig. 7B). The third are the *Obox1* and *Obox3* genes, which are required for oocyte maturation, ZGA and early embryogenesis [52].

### No correlation between DNA methylation and gene expression.

**Figure 7:**
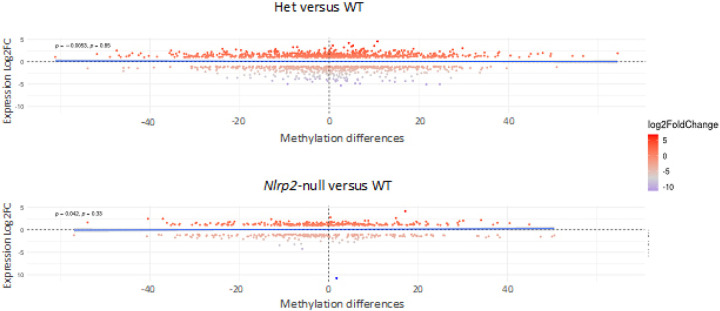
Scatterplots displaying CpG methylation and gene expression correlation. Each dot represents one DEG. X-axis: % CpG methylation difference of gene bodies. Y axis: Log2FC gene expression difference. Upper panel: Het to WT comparison; Lower panel: *Nlrp2*-null to WT comparison (*p*: Spearman’s rank correlation coefficient).

Given that transcription drives DNA methylation establishment within gene bodies in oocytes, we aimed to explore the relationship between gene expression and DNA methylation using integrative scatter plots that contrast the expression differences of DEGs with their corresponding gene body DNA methylation differences. There was no correlation for either the Het to WT comparison or *Nlrp2*-null to WT comparison (Spearman correlation coefficient of −0.0053, *p* = 0.85 for Het versus WT and spearman correlation coefficient of 0.042, *p* = 0.33 for *Nlrp2*-null versus WT; Fig. 7 upper and lower panel, respectively). To further investigate the correlation between gene expression and DNA methylation, we analyzed the 1370 DEGs from the Het versus WT comparison and the 526 DEGs from the *Nlrp2*-null versus WT comparison that also had available methylation data. We selected the top 25% of DEGs between Het and WT oocytes and plotted them against the top 25% of genes with the highest gene-body methylation differences (Supplementary Fig. 6A). This analysis yielded 66 DEGs (list 1 in Supplementary Table 13). Subsequently, we plotted the top 25% of DEGs between *Nlrp2*-null and WT against the top 25% of genes with highest gene-body methylation differences (Supplementary Fig. 6B). This resulted in a list of 37 DEGs (list 2 in Supplementary Table 13). This analysis yielded intriguing targets for potential future detailed analysis. List 1 included *Zbed3*, which encodes a member of SCMC, and *Setd1b*, which encodes a histone H3K4 methyltransferase required for oogenesis [53]. List 2 included *Uhrf1*, encoding a DNMT1 co-factor, *Tubb6*, encoding a tubulin involved in microtubule cytoskeleton organization and the mitotic cell cycle, and *Kdm1a/b*, encoding histone H3K4 demethylases required for the establishment of DNA methylation in mouse oocytes [7].

Finally, we found that oocyte gene expression differences resulting from absence or reduced *Nlrp2* are associated with only subtle DNA methylation differences, because only approximately 20% of DEGs between Het and WT (273 out of 1370) or DEGs between *Nlrp2*-null and WT oocytes (108 out of 526) had ≥ 20% gene body methylation differences. The absence of a strong correlation between gene expression and DNA methylation differences, including for genes that encode key factors that shape the oocyte methylome and transcriptome, suggests that the methylation differences between Het and WT oocytes and between *Nlrp2*-null and WT oocytes are likely not driven by altered transcription. This may be because identified DEGs in *Nlrp2*-null and Het oocytes predominantly result from post-transcriptional changes in transcript abundance from altered RNA stability and RNA degradation, rather than from changes in active transcription.

## Discussion

NLRP2 is a protein of the SCMC of oocytes and preimplantation embryos. Maternal NLRP2 function in oocytes is important for methylation and expression of selected imprinted genes in placentas and embryos or offspring in both humans and mice [21, 54–56]. Our previous studies in *Nlrp2*-null mice have shown that maternal complete loss of NLRP2 causes reduced fecundity, embryonic and placental defects, and offspring with birth defects and growth abnormalities, with evidence of methylation and expression changes at selected imprinted loci. These defects cannot be rescued by embryo transfer into the uterus of WT females [39], implying that they originate in oocytes. This is further supported by our observation that ovaries of *Nlrp2*-null females have altered follicle distribution [21], itself a feature that had not been previously reported for inactivation of other similar SCMC protein coding genes, such as *Tle6, Khdc3*, and *Nlrp5*, but investigations of oocyte and follicle morphology in those reports were more limited. This supports that NLRP2 has a broader role in oocyte epigenetic reprogramming and gene expression, with important consequences for pregnancy and offspring development. Although altered oocyte and early embryo epigenetic reprogramming has been shown in mouse oocytes and embryos lacking another SCMC protein, such as NLRP14 [57], this has not yet been examined for NLRP2. Although the reproductive phenotypes of maternal loss of *Nlrp2* are variable, there are currently no studies that have examined if these phenotypes are only observed with complete loss of maternally expressed SCMC proteins like NLRP2, or whether their dosage is important.

To this end we characterized how loss or a lower amount of NLRP2 in oocytes impacts their development, transcriptome, and methylome. We performed, to our knowledge, the first detailed morphological characterization of ovarian follicles of *Nlrp2*-null, *Nlrp2*-Het and WT females, followed by parallel transcriptome and methylome profiling using the G&T-seq protocol on isolated GV-stage oocytes from 26–28 days-old female mice of these genotypes. Confirming our previous data [21], we found altered follicle distribution in the ovaries of *Nlrp2*-null mice, with fewer secondary, antral and ovulatory follicles and more atretic follicles and we also noted that GV oocytes from *Nlrp2*-null mice tend to degenerate faster during isolation. Both observations indicate that the GV oocytes from the *Nlrp2*-null mice are developmentally compromised, even though all current data indicate that they are fertilization-competent in both human and mice, although with variable embryo development and pregnancy outcomes. What we did not expect, was that ovaries from Het females also have altered follicle distribution, since these mice are fertile and have no known adverse reproductive outcomes [21]. This was the first indication that not only loss, but also reduced *Nlrp2* negatively impacts oocyte development which, to our knowledge, has not been shown in any other mouse model with inactivation of genes encoding proteins of the SCMC. Interestingly, this may be relevant to data from some human studies showing that heterozygous maternal loss of *Nlrp2* and other SCMC proteins is associated with early pregnancy loss and other adverse reproductive outcomes [37, 42, 58–60].

This supported the importance of performing transcriptome and methylome profiling on oocytes from all three genotypes, Null, Het and WT. We selected the GV-oocyte stage for this experiment because it is the closest to the time that transcription and methylation reprogramming in oocytes is completed, making it less likely that any observed changes result from altered oocyte growth or maturation beyond this stage. Furthermore, collecting oocytes at this pre-ovulatory stage allowed us to collect enough oocytes from ovaries without the required need for superovulation to collect larger numbers of post-ovulatory oocytes. Superovulation recruits oocytes that are not primed for ovulation and therefore may not have completed epigenetic maturation. The effects of superovulation on gene expression have been demonstrated in some studies [61, 62]. Importantly, we wanted to avoid superovulation due to findings indicating a significant reduction in DNMT1 protein expression in mouse GV oocytes after exposure to hormonal treatments used in superovulation protocols compared to those not exposed to these treatments [61, 63]. Moreover, our previous study showed that superovulation of *Nlrp2*-null female mice results in more severe embryo phenotypes [21]. As for the morphological studies, we found that GV-oocytes of both Het and Null females have altered transcript abundance and methylation patterns compared to WT oocytes. This indicates that there is a mismatch between the morphological and molecular phenotypes of GV oocytes and the reproductive outcomes, since despite morphological and molecular changes, the oocytes of both *Nlrp2*-null and Het mice are fertilization competent, and in the case of Het oocytes, after fertilization yield pregnancies with comparable outcomes to WT. This suggests that the oocyte defects can at least be partially compensated for through a currently unknown mechanism.

Transcriptome profiling revealed two interesting findings. *First*, we found that Het and Null oocytes contained fewer expressed genes overall, possibly reflecting a shift in oocyte maturation. This coupled with impaired follicle maturation suggests that these animals are developmentally delayed. *Second*, there was substantial but incomplete overlap between Het and Null in genes with altered expression, that was more prominent among overexpressed genes. Notably, these common overexpressed DEGs contained genes that encode important epigenetic regulators, including ZFP57, DNMT1, UHRF1 and KDM1B. We also found some of the factors involved in oocyte transcriptome shaping among common overexpressed DEGS. Examples include CPEB1, and ZAR1/2. We further found that other proteins of the SCMC, including TLE6, OOEP, PADI6 and ZBED3 among the common overexpressed genes. Since transcriptome analysis by RNA-Seq merely reflects abundance of RNAs in the oocytes, which are transcriptionally quiescent by the GV stage, we propose that altered RNA abundance could either reflect overexpression at an earlier stage, increased abundance due to increased transcript stability, delayed clearance of maternal transcriptome, or a combination of these. This could be related to changes in cytoplasmic lattices (CLs) in Het and *Nlrp2*-null oocytes. It is well established that oocytes store mRNAs and proteins that are essential for embryonic development, which includes the regulators of the epigenetic reprogramming that takes place in the early preimplantation embryo after fertilization and before the embryo activates its own biparental genome. These proteins localize to CLs, which are twisted fibers of individually stacked filaments, and of which the SCMC is a component at the cell’s periphery [64]. The presence of an intact SCMC is vital to the integrity of the CLs and for preventing premature degradation or premature nuclear localization of stored proteins and consequent early embryo arrest [22, 27, 30, 31]. If the disrupted CLs cause altered protein abundance in the cell, the expression changes could be to compensate for lost or mislocalized proteins, for example increased shuttling into the nucleus. We have shown that loss of maternal NLRP2 alters cytoplasmic localization of DNMT1 [21]. Knockout models of *Padi6, Nlrp14* and *Stella* [44, 57, 65, 66] also have mislocalized DNMT1 along with its co-factor UHRF1, with aberrant nuclear localization of the DNMT1/UHRF1 complex, coupled with global hypermethylation and impeded ZGA [57, 65, 66]. DNMT1 is a known maintenance methyltransferase [67], but its characteristics support that it can also function as a de novo methyltransferase [65]. This may in part explain the altered methylation seen in *Nlrp2*-null and Het oocytes.

There were also several interesting findings in the methylation profile data. *First*, genome-wide DNA methylation levels of Het and Null oocytes were not significantly different from those of WT oocytes. However, compared to WT oocytes, Het and Null oocytes had significantly lower methylation levels at promoters (1.6% and 2.1% versus 3.6%), and at gene bodies between Het and WT (43.5% versus 41.8%), but not between Null and WT. *Second*, when we examined imprinted gDMRs, we confirmed high methylation of maternal gDMRs and low methylation of paternal gDMRS, with no difference between genotypes. Similarly, when examining 11,720 CGIs with sufficient sequence coverage, we found that only15 had significantly altered methylation, 6 between Het and WT and 9 between Null and WT (Supplementary Table 10), and that none of the 2140 highly methylated oocyte-specific CGIs had different methylation between genotypes. These observations are consistent with those in oocytes of *Padi6* and *Nlrp14* knockout mouse models [44, 57], and were predictable since any significant change in methylation of these features would be expected to have more drastic phenotypic consequences. Among the genes controlled by CGIs with altered methylation, were *Fzd5* (essential for yolk sac and placental angiogenesis [68]), *Hfm1* (mutations are linked to decreased embryo development capacity and increased number of cell division times[69]), *Parp8* (required for spindle positioning to oocyte cortex and mediating asymmetric divisions [70]), *Fgfr2* (crucial for trophectoderm development and blastocyst implantation [71]) and *Tcf3* (required for proper axial mesoderm structures in the early embryo [72]). *Third*, when we examined the larger hyper and hypomethylated domains characteristic of oocytes, known as HyperDs and HypoDs, we found that quantitatively, the overall bimodal methylation patterns were well conserved across genotypes. However, a deeper analysis of distinct domains indicates that a subset of the HyperDs lost methylation in Het and Null oocytes and conversely, a subset of the HypoDs gained methylation in Het and Null oocytes. When we agnostically examined regions with differential methylation between the genotypes using a 10-kb tiling approach, we found 378 hypomethylated and 599 hypermethylated DMRs in Het compared to WT oocyte pools and 907 hypomethylated and 1393 hypermethylated DMRs in *Nlrp2*-null oocytes compared to WT oocytes. We noted that compared to WT, *Nlrp2*-null and Het gained DMRs in intergenic regions than in promoters, exons, or introns. None of these observed methylation changes favored any specific chromosomes, indicating that there are genome-wide imbalances in DNA methylation in both Het and *Nlrp2*-null oocytes. It remains to be determined whether this is causally related to the mislocalization of DNMT1 or other epigenetic regulators that influence DNA methylation.

The final important step of our analysis was the integrative analysis of transcriptome and methylome, to begin to shed light on whether altered transcription was at the origin of the methylation changes in the Het and Null oocytes. The lack of correlation indicates that this is likely not the case and that the methylome and transcriptome are disrupted through independent mechanisms in these mutant oocytes.

## Conclusion

Our study reveals a significant role for the maternal effect gene *Nlrp2* in shaping the transcriptome of GV oocytes, likely through a mechanism unrelated to DNA methylation establishment. We also showed that not only its absence in *Nlrp2*-null animals but also its reduced dosage in Het animals result in morphological, gene expression and methylation differences in GV oocytes. Furthermore, given the impaired follicle maturation and overall lower number of expressed genes in Het and *Nlrp2*-null oocytes, it is possible that these cells are developmentally delayed compared to WT oocytes. Thus, the adverse reproductive outcomes of *Nlrp2*-null, and to a lesser degree, Het mice maybe be caused by a combination of altered gene expression, delayed oocyte development and altered epigenetic programming, but considering we found no evidence of a correlation between gene expression and DNA methylation changes, how these are mechanistically connected remains to be further studied.

## Materials and Methods

### Oocyte collection and biological replicates

All experiments were approved by the Institutional Animal Care and Use Committee (IACUC) at Baylor College of Medicine (BCM) under study protocol (AN-2035) and conducted according to institutional and governmental regulations concerning the ethical use of animals. Animals were housed in facilities approved by the Association for Assessment and Accreditation for Laboratory Animal Care International (AAALAC).

We collected fully grown germinal vesicle (GV) oocytes from the ovaries of our previously generated *Nlrp2*^*tm1a/tm1a*^ *(Nlrp2-null), Nlrp2*^*tm1a/+*^ heterozygous (Het), and *Nlrp2*^*+/+*^ (WT) virgin females at 26–28 days of age [21]. To avoid any transgenerational reproductive effects in Het females from a maternally inherited null allele, we only studied Het females that were offspring of crosses between *Nlrp2*^*tm1a/+*^ females and *Nlrp2*^*tm1a/tm1a*^ males. We mechanically released oocytes from ovaries using a fine needle [73] and denuded recovered oocytes from granulosa cells by gentle pipetting with 75 *μ*m stripper tips (CooperSurgical, Inc, USA) in M2 medium (Sigma, Japan). We then washed clean oocytes twice in PBS and collected 60–80 oocytes from both ovaries combined from each female, which served as one biological replicate oocyte pool. We lysed oocyte pools in 5 μl RLT Plus buffer (Qiagen, Germany) and stored them at − 80°C until further use.

### Histological Studies

Ovaries were collected from three week-old mice, dissected from surrounding fat in 1X PBS and fixed in Formalde-Fresh (Fisher Scientific, Pittsburg, PA) overnight at 4°C. Fixed tissues were washed twice for 30 minutes in 1X PBS and subsequently dehydrated in 50%, 70% ethanol, and 3x in 100% ethanol, for an hour each at room temperature (RT). The tissues were then treated 3x for 30 minutes each with xylene, followed by 2× 1 hour in paraffin at 55\varvec°C before embedding tissues in paraffin. Paraffin-embedded tissues were then serially sectioned at 5–8 μM thicknesses and used for hematoxylin and eosin staining (H&E) and microscopy to assess oocyte and follicle morphology. Every 10th section was photographed and the number and developmental stages of follicles containing oocytes within each section were recorded. Five females from each genotype *(Nlrp2*-null, Het and WT) were used for the analysis.

### Parallel bisulfite and RNA sequencing by genomes and transcriptomes sequencing (G&T-seq)

To perform parallel bisulfite and RNA sequencing from the same thawed oocyte-pool lysate, we completed the initial 53 steps of the published G&T-seq protocol with minor changes to obtain gDNA and RNA from each oocyte pool [74]. Purified gDNA was then used for BS conversion and PBAT library preparation according to the described protocol [75] with minor changes. We first prepared MyOne Streptavidin C1 Dynabeads (Fisher Scientific, USA) according to manufacturer’s instructions, labeled them with biotinylated oligo-dT30VN (100 *μ*M) to make the RNA libraries. We then resuspended the oligo-dT30VN labeled Dynabeads into bead resuspension buffer before adding them to the tubes containing the cell lysates. We placed the tubes on a magnet to physically separate polyadenylated mRNA attached to the beads and gDNA in the supernatant. We transferred the supernatant containing the gDNA to a new tube and washed the Dynabeads three more times in wash buffer to maximize the gDNA recovery. To make the RNA-Seq libraries, we reverse-transcribed the polyadenylated mRNA attached to the Dynabeads and PCR-amplified the obtained cDNA before tagmentation-based cDNA library preparation with Illumina’s Nextera XT Index Kit v2 Set C per the manufacturer’s instructions. Briefly, we subjected 200 picograms of amplified cDNA underwent tagmentation at 55°C for 5 min and mixed them with 1 *μ*l of index 1 (one of S513, S515, to S518, S520 to S522) and 1 *μ*l of index 2 (one of N701 to N707, N710 to N712, N714, N715). We then PCR-amplified and purified these products on Agencourt AMPure XP beads (Beckman Coulter, USA) in a 0.7:1 volumetric ratio and eluted them in nuclease-free water.

To make the PBAT libraries, we purified the gDNA with Agencourt AMPure XP beads in a 0.9:1 volumetric ratio to capture gDNA fragments of > 200 bp. We then bisulfite-converted the recovered gDNA using the EZ Methylation Direct Kit (Zymo Research, USA) per manufacturer’s instructions and double-tagged the converted DNA with Illumina adapters. To add the 6NF oligo or Preamp Oligo (IDT, USA) which acts as Illumina adaptor one to the bisulfite-converted DNA, we eluted the gDNA fragments in 40 *μ*l of first-strand synthesis master mix purified this first-strand synthesis product in a 0.8:1 volumetric ratio of Agencourt AMPure XP beads (Beckman Coulter, USA). We then added 40U of exonuclease I (New England Biolabs, USA) to the reactions for exonuclease digestion and eluted this product in 50 *μ*l of second-strand synthesis master mix to add the 6NR oligo or Adaptor 2 Oligo (IDT, USA) (which acts as Illumina adaptor two) by second-strand synthesis. We purified this product in a 0.8:1 volumetric ratio of Agencourt AMPure XP beads (Beckman Coulter, USA). Both amplifications were mediated by 50U of Klenow exo- (Enzymatics, USA). We next added a 50 *μ*l PCR master mix with the Illumina forward PE1.0 primer and Illumina reverse indexed primer (iPCRTag) to the beads to amplify the libraries using 1U of KAPA Hifi Hotstart DNA polymerase (Roche, Switzerland). We then purified the libraries with a 0.8:1 volumetric ratio of Agencourt AMPure XP beads (Beckman Coulter, USA) and eluted them in EB buffer for storage at −20° Cuntil sequencing. The composition of all master mixes and programs used for amplifications are shown in supplementary Tables 14 and 15 respectively. All primer sequences are listed in supplementary Table 16.

### Data processing

#### RNA-seq data processing and differential expression analysis

We conducted analysis on a total of 17 libraries. Quality Control (QC) of the raw reads was performed using FastQC [76], followed by Read Processing of adapters and quality trimming using Trim Galore [77]. Trimmed sequences were aligned to the Genome Reference Consortium mouse genome build 38 (GRCm38) with HiSat2 version 2.1.0 [78] in single-end mode. The data were quantified in SeqMonk and analyzed in Rstudio. We excluded reads aligning within unknown non-canonical splice junctions, and quantitated RNA-seq data by counting reads crossing known and annotated splice junctions in the gene by building a canonical_splice_count.py tool. To run the program, we provided BAM files from a spliced alignment program (HiSat2) and a GTF file of gene annotations (GRCm38). As the program necessitates an exact match between observed splice and the position of the intron, we provided the same GTF file to the aligner when running the alignment. The program generates two output files: a Stats file, the details of which are presented in Supplementary Table 3, and a raw count matrix file suitable for input into DESeq2. Briefly, a differential expression analysis was conducted using DESeq2 version 1.42.0 [79]. Genes were considered differentially expressed using a Wald test and based on a threshold of an adjusted P-value < 0.05 and |log^2^FoldChange| > 1. We additionally utilized the plotMA function to showcase the log2 fold changes associated with the mean of normalized counts for all the genotypes in the dataset.

#### PBAT data processing and differential methylation analysis

Methylation sequencing data were processed using the standardized and reproducible processing pipeline nf-core/methylseq version 1.6.1 [80] [81] inside of docker containers orchestrated by the workflow manager nextflow version 23.11.0 [82]. Briefly, the processing pipeline consisted of sequencing Read QC using FastQC [76] followed by adapter sequence trimming using Trim Galore [77]. Trimmed reads were then aligned to the pre-indexed reference genome mm10 using bowtie2 [83]. Read alignments were then deduplicated and methylation calls were extracted, reported, and converted into coverage2cytosine reports using Bismark [84] with the –pbat flag parameter, followed by Alignment QC using Qualimap [85]. All logs, summary tables, and quality control reports were agglomerated into a final report using MultiQC [86]. Coverage2cytosine reports were then imported into R version 4.3.0, converted into a methylRawList object using a 1x minimum coverage threshold cutoff using methylkit [87]. Downstream analyses were conducted using methylkit and consisted of filtering by Coverage (lo.count = 1, lo.perc = 0.1, hi.perc = 99.9), Pearson correlation comparisons within treatment groups, ascertaining percent methylation for PCA analysis, and calculating differential methylation statistics between groups using either logistic regression test or Fisher’s Exact test with McCullagh and Nelder correction for overdispersion and adjustment of p-value significance using Benjamini-Hocherg multiple test correction.

## Data Availability

Data is provided within the manuscript or supplementary information files.
